# Improving the health of people with intellectual disability: past progress and future directions

**DOI:** 10.3389/fpsyt.2026.1804423

**Published:** 2026-04-21

**Authors:** Gloria L. Krahn, Julian N. Trollor

**Affiliations:** 1 College of Health, Oregon State University, Corvallis, OR, United States; 2 2National Centre of Excellence in Intellectual Disability Health, UNSW Medicine & Health, University of New South Wales, Sydney, NSW, Australia

**Keywords:** data linkage, disability competencies, health, health disparities, intellectual disability, progress

## Abstract

Over the past decades substantial progress has been made in addressing health disparities of people with intellectual disability. The “era of awareness” (pre-2005) identified a “cascade of disparities” of more adverse health conditions, less attention to health needs, poorer health care access, and fewer opportunities for health improvement. This occurred alongside emerging governmental recognition of the poor health of people with intellectual disability. The “era of assessment” (2005-2015) saw advances in improved health data, population health surveillance, attention to environmental contributors, and passage of the Convention on the Rights of Persons with Disabilities. Research extended from clinical samples to population-based studies with greater focus on social determinants of health. The “era of context” (2015-2025) was characterized by the COVID-19 pandemic, technology, and greater inclusion of people with intellectual disabilities in their communities. Data advances included a disability data framework, clearer disability definitions and identification, and greatly expanded data-linkage research to inform programs and policies. Disability health competencies were developed in multiple countries for multiple disciplines. For the coming decade, we anticipate greater use of technology, especially Artificial Intelligence, expansion of the “inclusion movement,” and attention to ableism. Data linkage and analysis in multiple countries will map longitudinal health trajectories. We anticipate a much-needed resurgence in attention to mental health. Global climate change will demand inclusive emergency preparedness. Future advances require that research, health care, service programs, and communications be co-designed with people with intellectual disability and their supporters. Focus and funding are critical through investment proportionate to need.

## Introduction

Poor health among people with intellectual disability has been recognized for decades, with a substantial but undetermined portion considered preventable disparities (1–6). Although the persistent reports of health disparities might suggest little advance, the authors believe significant progress has been made in identifying, measuring and improving the health of people with intellectual disability. Meaningful changes, both minor and major, typically occur step by step, with advancement building on previous progress both within the health domain, and in conjunction with broader, cross-sector transformations. This paper reviews advances over the past thirty years, summarises our perspectives based on a broad review of the literature and our knowledge of developments, highlights areas requiring more rapid progress, and suggests future directions to advance health care for people with intellectual disability. The authors write from positions as academic researchers in the U.S. and Australia. The first author has experience in U.S. federal government service and public health programs and policies; the second has engagement with Australian health services, regulatory bodies and policy. The paper is limited to English-language publications and primarily high-income countries.

## Step-by-step progress in the recent past

### Era of awareness (to 2005)

#### National reports

From 2001 to 2005, a number of countries including the UK (7), USA (8), Scotland (9), Australia (10), and Canada (11) issued national reports highlighting poor health among people with intellectual disability. These reports focused on early detection, reducing comorbidities, and empowering individuals and carers to improve health outcomes (12). In 2001, the World Health Organization published the International Classification of Functioning, Disability and Health (13) which for the first time, identified environmental factors alongside personal factors and health condition as influencing health and function. This further promoted incorporation of the social model of disability into understanding health.

#### A cascade of disparities

A 2006 review (12) identified that much of the previous literature related to unique health needs of clinical samples, finding under-diagnosis of health conditions (14, 15), over-prescription of anti-psychotic drugs, and diagnostic overshadowing (15, 16). People with intellectual disability experienced “a cascade of disparities,” with each disparity compounding the effects of others. First, they experienced more adverse health conditions including epilepsy, heart conditions, mental illness, poor oral health, vision and hearing problems (2, 16–20). While some of these problems related to the disability etiology (e.g., Down syndrome), others were preventable. Second, they experienced disparities in attention to their health care needs, due to communication differences, behavioral concerns, inconsistency in supports, and delays in seeking health care (15, 21–24). Third, the literature documented disparities in access to quality health care related to clinicians’ lack of training and expertise, and difficulties with logistics like transportation (23–25). Finally, people with intellectual disability experienced disparities in preventive care and health promotion, with fewer opportunities to learn how to improve health behaviors and adopt healthier lifestyles (15, 16, 26, 27). This cascade of disparities led to poor health outcomes.

#### Self-determination

This period also witnessed growth in the self-determination and person-centered planning movements that had been initiated in the 1980’s. Advocacy groups implemented curricula to teach self-determination skills to adolescents and adults with intellectual and developmental disabilities, and self-directed supports became a guiding principle for service delivery (28). Clinical and community researchers developed and tested a number of health intervention tools. Examples included the ASK toolkit to promote self-advocacy for better health care (29), the CHAP checklist for general practitioners to attend to all relevant areas of health (30), and evidence-based curricula on health and healthy lifestyles for people with intellectual disability (31, 32). While this period was characterized by greater attention within the disability community to preventable poor health, the need persisted in broader policy and service systems for increased awareness and improvement.

### Era of assessment (2005-2015)

The next decade marked dramatic improvements in assessing the health disparities of populations with intellectual disability. Data-driven decision-making for the general population demanded the same approach for people with intellectual disability, resulting in calls for action to address disparities (33, 34). Attention shifted from conditions unique to people with disabilities to comparing their health with the general population using health indicators. The POMONA project (35–37) showed that health of people with intellectual disability could be assessed using 18 common health indicators across 13 European countries, filling a critical measurement gap and revealing a consistent burden of preventable conditions, lifestyle risk, and care‐quality shortfalls across Europe.

#### Role of environment

Environmental factors identified in the ICF framework gained much more attention, especially Social Determinants of Health (SDOH) SDOH frameworks were published by WHO (2008) (38), the European Commission (2009) (39), UK (2010) (40), Australia (2010) (41), USA (2011) (42), Canada (2015) (43) and Taiwan (2016) (44). Some frameworks focused on “causes of causes” such as macro-economics, early child development, and social policies (45), while others were more individual-focused covering economic stability, education, health promotion and health care, neighborhood and built environment, and social and community context.

#### Changes in foci of interest

**Figure 1:**
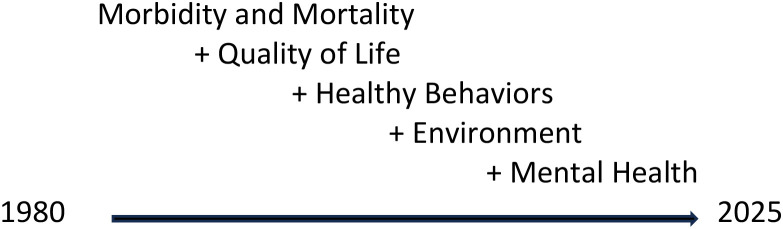
Health indicators over time for people with intellectual disability. Morbidity and Mortality + Quality of Life + Healthy Behaviors + Environment + Mental Health 1980-2025 Alt Text: list of how health indicators were expanded over time.

As indicated in **Figure 1**, over time, health indicators broadened from morbidity and mortality to include quality of life measures (e.g., self-reported well-being, self-determination, life satisfaction), health behaviors (e.g., smoking, nutrition, and physical activity), environmental factors, and subsequently, mental health (46–48).

#### Improved health surveillance

This decade experienced major improvements in disability health data and surveillance and a shift from clinical samples to population level surveillance. The United Kingdom’s Learning Disabilities Observatory (~2010-2020) published key population studies (49) and identified the “transition cliff”, a precipitous drop in prevalence in administrative data between childhood and adolescence to early adulthood (50). Use of health services research with administrative datasets revealed disparities such as more hospitalization for ambulatory care conditions like diabetes for people with intellectual disability (51).

#### Linked data

Between 2005–2015, the use of large, linked datasets and advanced “big data” capabilities emerged in several countries. Routine digitization of clinical and administrative data and strengthened stewardship enabled integrated linkages and analyses of factors affecting health outcomes. In Canada, Manitoba’s unique integrated data repository allowed investigation for people with developmental disabilities (34, 52), while in Australia, Leonard’s research group used intellectual disability registry data with other data sets to study the health of people with intellectual disability identified from birth onwards (53, 54). Governance advanced through privacy‐preserving linkage, controlled output disclosure, and audit trails. Despite progress, challenges with data completeness, coding fidelity and sample bias remained, underscoring the need for careful interpretation.

#### Chronic conditions

Marking a shift from conditions unique to people with disabilities, chronic conditions emerged as major contributors to poor health of people with cognitive disabilities. People with physical, cognitive or intellectual disabilities were documented to be at much higher risk for a number of chronic conditions, including diabetes and cardiovascular disease, and to experience them at earlier ages than the general population (55–58). Recognition of multimorbidity as a major contributor helped refocus the understanding of poor health of people with intellectual disability.

### Era of context (2015-2025)

#### COVID-19

The COVID-19 pandemic was the major health event of the decade, disproportionately affecting people with intellectual disability due to higher susceptibility to infection and death (59–61). This likely related to group living, personal care from itinerant providers, and higher rates of pre-existing health conditions. People with intellectual disability likely also experience more long COVID, though research is limited (62). The pandemic spurred calls for improved health data (63), telehealth and online supports (64), and remote work and socializing--changes that benefited some but also led to isolation and anxiety (65).

#### International reports

During this decade, the United Nations released the Flagship Report on Disability and the SDGs (66, 67) that, for the first time, included disability in the economic goals for development. WHO developed the Global Report on Health Equity for Persons with Disabilities (68) that brought attention to systemic contributors to health inequity, documented evidence across countries, and made recommendations for country-level actions with an initiative in 2025 to implement recommendations (69).

#### Improved health data and surveillance

Over the past decade, health data capabilities progressively matured in some countries. Notably, secure analytic platforms provided controlled access to linked unit-record data. Research adopted robust causal methods and transparent reporting. In Australia, this progress was underpinned by Australia’s National Digital Health Strategy 2023–2028. The Australian disability data framework (70), developed with input from people with lived experience, clearly defined necessary health data, current availability, and further data needs, serving as an international model. Aitken and colleagues (71) reported on various methodologies for constructing disability identifiers to link administrative datasets. Investments in large, linked data assets have improved researcher access, with national exemplars being England’s National Health Service (NHS) Learning Disability Data Hub (72), and Australia’s National Disability Data Asset (NDDA) (73) and National Health Data Hub (NHDH) (74). In Canada, where health care and other services data are provincially managed, a national (75) and some provincial datahubs (76–78) enable secure environments with access for researchers. In contrast, the USA lacked extensive health data linkages. Instead, the focus was on standardizing operational definitions for intellectual and developmental disabilities in administrative data to improve interpretability and use (79) (80, 81), ultimately supporting emerging efforts at data-linking.

Population-scale data linkages led by the second author’s team, enabled Australian studies on health service use, costs, comorbidities, and key health outcomes for people with intellectual disability (82–85). Analyses revealed significant gaps in life expectancy (86) and higher rates of potentially avoidable mortality (87). These findings informed interventions and shaped Australian policy to address health inequities.

National roadmaps to enhance health data for individuals with intellectual disability include routine identification in electronic health records and expanded data capabilities and comprehensive outcomes reporting in Australia (88). In the USA, data improvements call for the identifiers in national health surveys, standardizing analytic methods, increasing state research and analytic capacity, and improving communication about prevalence, health needs and health (89).

#### Mental health

Previous work in intellectual disability mental health identified the need for training (90–92), and this decade brought substantial advances in documenting and responding to the high mental health needs of this population (93, 94). Population level data linkages demonstrated elevated rates of mental illness (95), ineffectiveness of mainstream services (84, 96), and heightened vulnerability during key life transitions, including markedly increased dementia risk (95). Investment in targeted mental health initiatives remained limited, however, with few large-scale programs to build mainstream service capability or expand specialist care. Exceptions were the non-profit National Association for Dual Diagnoses in the USA (91), emerging programs in Queensland (97), New South Wales (98) and Victoria (99) in Australia; and sustained commitments in the NHS in England (100). Mental health services remain a gaping need for persons with intellectual disability.

#### Technology

The internet and social media greatly expanded opportunities for people with disabilities. Mobile apps enhanced accessibility for people with intellectual disability or limited literacy. For example, apps simplified text, simplified platforms, and supported information access and research participation for people with intellectual disability (101, 102). Advances in artificial intelligence (AI) began to reshape accessible information production for people with intellectual disability by coupling automated language simplification, inclusive design work-flows and assistive reading technologies. AI-enabled Easy Read authoring platforms enabled rule-guided simplification with context appropriate imagery across print, web and audio formats such as Photosymbols’ EasyMaker (103), while symbol-based environments streamlined creation of social stories, and visual timetables such as Widgit Online (104). Reader aids provided rapidly adjustable text, read-aloud and translation features without replacing Easy Read design (105, 106). These rapid developments await rigorous testing and require oversight and monitoring for mitigation of bias in sensitive domains.

## Anticipated directions for the future to 2035

### Era of inclusion

We are optimistic that dramatic progress in health of people with intellectual disability can be achieved over the next decade. Advances require focus and funding; both have been critical in the past to build infrastructure and fund research and programs. Keys to immediate progress include addressing ableism in health care and public health; longitudinal research; emphasizing integrated, person-centered approaches; supporting data-enabled health ecosystems with inclusive governance; harnessing technology, including AI; improving workforce capabilities; and inclusive planning and management for disasters (see **Table 1**).

**Table 1:** Anticipated directions and recommendations for the future decade to improve health of people with intellectual disability.

Anticipated direction	Recommendations for full and equitable impact
Inclusion and Ableism	• Include people with intellectual disability and their carers in planning, implementation and monitoring of all health services and programs affecting them • Develop tools and incentivize their use by health care professionals, service providers, and researchers to self-examine and self-monitor for ableism • Mandate ableism training for professionals and service providers, developing additional resources as needed
Workforce capability and systems change	• Train across disciplines for health competence in serving people with intellectual disability • Ensure leadership roles in workforce development and educational initiatives for people with intellectual disability
Integrated, people-centered care and services	• Examine health systems for discriminatory practices, develop improvement plans, and monitor implementation and effectiveness • Develop and rigorously evaluate approaches and technologies that improve cross sector and stakeholder collaboration in health care
Data-enabled ecosystems with safe, inclusive governance	• Ensure interoperable disability flags in all mainstream health and human service systems to improve visibility of population health outcomes • Develop accessible, interactive health outcomes dashboards for use by people with disability, services and policy makers; ensure minimum delays in data feeds • Develop methodologies to support enhanced inclusion of people with intellectual disability in population health data research and monitoring
Technology and artificial intelligence	• Determine the priorities and perceived utility of technology and AI from the viewpoint of people with intellectual disability and their supporters • Include people with intellectual disability in planning and implementing new technologies
Resilience to climate-related emergencies and other disasters	• Assess the specific vulnerability of people with disabilities to climate change and conduct co-designed research to inform mitigation strategies • Include people with intellectual disability and carers in preparedness planning, disaster management, and post-event monitoring and recovery

#### The power of together—inclusion and ableism

The intent of the UNCRPD (107) is increasingly being realized in many countries, though less so in the USA which has not ratified CRPD. We anticipate inclusive approaches in research (108–110), disaster planning (94), and communication will become more routine and refined. However, inclusion is revealing rampant ableism, including among health care professionals, service providers and researchers. Research documenting ableism (111–113) is calling us to consciously alter our assumptions and behaviors. Much like sexism and racism, reducing ableism will take time but be transformative for the health and wellbeing of people with intellectual disability. To address previous lack of exposure in curricula, we advocate for routine bias training for all health staff, grounded in lived experience.

#### Lifecourse approach and longitudinal studies

A lifecourse approach seeks to understand the health trajectories of persons with ID by identifying life-tasks and critical events at different ages and identifying relationships among outcomes over time. Swanson’s conceptual model developed for children with spina bifida (114) has been extended to older populations with intellectual disabilities (115). Two longitudinal studies, IDS-TILDA study in Ireland and HA-ID in the Netherlands have followed cohorts of aged individuals with ID over multiple timepoints, documenting longitudinal changes in health, social circumstances, and mortality (55, 56, 116, 117). Alternatively, linking administrative data sets is providing another view into longitudinal changes (118, 119). We anticipate greater use of lifespan approaches and longitudinal designs in the future.

#### Health workforce capability, practice standards and health system change

To effect improvements, the future health workforce must possess the confidence, skills, and experience to deliver tailored health care and public health programs for people with intellectual disability. Following a specialty model, the Netherlands initiated a 3-year specialty training of ID physicians in 2000 and subsequent guidelines for adolescent transition of care (120), and the UK and Ireland developed ID nurse specialists (121, 122). Following an inclusion model, some jurisdictions such as the U.K. mandate intellectual disability training for the health and social service workforce (100), while in others, professional development depends on individuals proactively seeking out opportunities. Australia and Canada provide comprehensive capability development resources for health professionals (123–125), while in the USA professionals have developed competencies for health (126) and public health professionals (125). To ensure meaningful impact, intellectual disability content should be mandated in all health professional continuing education curricula.

However, disability‐competent practitioners cannot improve outcomes without supportive health systems. Systemic barriers persist, including discrimination in accepting patients, inadequate appointment lengths, poorly adapted communication or intervention programs, challenges in finding and referring to specialty services, and policies that directly or indirectly limit access to medical equipment, vaccines, treatments, and organ transplants. As systems face greater accountability for population outcomes, they must identify and address systemic barriers.

#### Strengthening integrated, people-centered care and services for people with intellectual disability

The WHO’s Fourteenth General Programme of Work (GPW14) frames a threefold mission—promote, provide, protect—that links social determinants, strong primary health care, and system resilience (127). This vision will only be realized if accountability measures for equity and key performance indicators important to people with intellectual disability are included in public health and primary and preventive care. People with intellectual disability need to be factored into all broader health systems transformations. For people with intellectual disability, responsive systems require supportive decision-making, accessible communication, continuity of care across transitions, and reasonable adjustments embedded in hospital and health care policies and standards. Routine use of patient reported outcome and experience measures is pivotal to understanding what matters to people with intellectual disability. National health care regulatory standards need actionable measures that raise health care quality, reduce harm, and improve outcomes. Aligning service models with WHO’s integrated, people-centered services will promote care that is co-produced, culturally safe, and coordinated across the life course (127).

#### Data-enabled ecosystems with safe, inclusive governance

Delivering and monitoring equitable improvements for people with intellectual disability requires cross-sectional and longitudinal analytics with adequate privacy protection and accessible reporting. Jurisdictions will need to prioritize linkage between disability, education, social services, health services, and mortality datasets. Data fields need to include SDOH, hospitalization, primary care (including health checks), medicines, immunization, cancer screening, chronic conditions and cause of death, to ensure adequate capture of outcomes. Routine cohort and subgroup reporting, including all major diversity characteristics, is important to understand the distinct needs of this heterogeneous population. As single source ascertainment of disability status within administrative data is inadequate (128), a key challenge is uniform adoption of disability-specific indicators across administrative datasets. Indigenous data sovereignty principles must guide all analyses and public reporting in Australia (129, 130); in the USA and elsewhere, data analyses should disaggregate by race, ethnicity and other marginalized identities (131).

#### Technology and artificial intelligence

Advances in AI can expand access to health information for people with intellectual disability by transforming both content and delivery of communication. Carefully governed generative language models with visual supports and multilingual options can automatically produce Easy Read versions of health materials at point of need such as procedure preparation, appointment letters, care plans, emergency preparation information. Inclusive chatbots will provide accessible, cited health information using speech/AAC and iconography, with safeguards that escalate to human support as needed. While accessible explanations can be generated automatically, clinician review will remain essential for accuracy and inclusive decision making.

To realize full impact, these future systems must be co-designed with people with intellectual disability and their supporters to ensure readability, acceptability, truthfulness, actionability, and scalability. Access to technology will need to be ensured for low-income groups to reduce the technology-access gap and realize equitable improvements. Finally, future health navigator models are needed that combine human support with AI-enabled digital coordination (appointments, reminders, transport, benefits, cross-sector referrals) to reduce fragmentation across general practice, specialist care, allied health, and disability services.

#### Resilience to climate-related emergencies and other disasters

As part of the global population, people with intellectual disability will face more frequent disruptions, with substantial impacts on safety, health and wellbeing (132). WHO’s GPW14 embeds climate risk and emergency preparedness into system planning (127), and jurisdictions must prepare for those most vulnerable. This requires inclusive preparedness planning (e.g., evacuation or sheltering in place), support during events (e.g., access to needed equipment, vaccines, or medications), and post-event recovery supports (133). Equity-sensitive emergency planning must include people with intellectual and other disabilities, and provide accessible alerts, backup virtual care, secure medication and equipment supply chains, and proactive welfare checks. Co-design and strong cross-agency linkages, for example between disability and health services, should be standard in emergency preparedness.

## Conclusion

Although health disparities continue for persons with intellectual disability, significant progress has been made in awareness of the need, improved methods for measuring and understanding the reasons for preventable poor health, and recognition of the impact of environmental contributors to poor health. Attention to ableism, longitudinal research, improved capability of health work forces, integrated services, expanded use of data linkage, technology, and inclusive disaster planning are anticipated to be key directions for the coming decade. Focus, funding and inclusion of people with intellectual disability and their families is essential for real progress in the future.

## Data Availability

The original contributions presented in the study are included in the article/supplementary material. Further inquiries can be directed to the corresponding author.
